# Clinical and research implications of the findings of the Tanzania 2022 Population and Housing Census for the development and suitability of neuropsychological tests for older adults in Tanzania

**DOI:** 10.1186/s12877-025-06071-9

**Published:** 2025-05-30

**Authors:** Damas Andrea Mlaki, Victor Valcour, Andjelika Milicic, Elaine Allen, Stella-Maria Paddick, Aaron Berkowitz, Raina Kiama, Bruce Miller

**Affiliations:** 1 Global Brain Health Institute, University of California San Francisco, Suite 3C08, Weill Neurosciences, 1651 4th Street, Box 3020, San Francisco, CA 94143 USA; 2 Department of Epidemiology and Biostatistics, University of California San Francisco, 550 16th St., San Francisco, CA 94158 USA; 3 Campus for Aging and Vitality Newcastle University, Westgate Road, Newcastle upon Tyne, NE4 6BE UK; 4 Directorate of Medical Services, Mirembe National Mental Health Hospital, Hazina, Dodoma 41108 Tanzania; 5 National Bureau of Statistics, 64 Lusinde Road, Tambukareli, Dodoma, 41104 Tanzania

**Keywords:** Neuropsychological tests, Older adults, Literacy, Census, Clinical, Research, Tanzania

## Abstract

**Background:**

The identification of dementia using valid and reliable neuropsychological tests is crucial for the development of effective preventive interventions, treatments, and care plans.

**Methods:**

We analysed the 2022 *Population and Housing Census* data in Tanzania to determine the age-adjusted prevalence of subjective memory, hearing, and visual complaints and explore factors that may influence the development and suitability of neuropsychological assessment batteries among adults aged 60 and older. Age-adjusted prevalence estimates were calculated using the WHO Direct Method. Logistic regression models were performed to examine the factors associated with memory complaints. Mediation analysis was conducted using path analysis, and the significance of the indirect effects was tested using bootstrapping procedures.

**Results:**

Adults aged 60 + constituted 5.7% of the population. The median (IQR) age was 68 (60–97) years. Literacy and numeracy rates were 59.7% and 67.7% respectively. The age-adjusted prevalence of subjective memory, hearing, and visual complaints, were 7.3% (95%CI: 7.2–7.4), 7.5% (95%CI: 7.4–7.6), and 16.3% (95%CI: 16.2– 16.4). Being married (AOR 0.83; 95%CI: 0.74–0.93; *p* = 0.002) and having a seven-year education or more (*p* ≤ 0.001) reduced the odds of memory complaints, while hearing (AOR = 4.62; 95%CI: 4.37–4.88; *p* ≤ 0.001) and visual (AOR 7.12; 95%CI: 6.78–7.47; *p* ≤ 0.001) complaints increased the likelihood of memory complaints. Age (*p* ≤ 0.001) and female sex (*p* ≤ 0.001) accounted for 21% and 7% of the effects of sex and education on subjective memory complaints respectively.

**Conclusion:**

Literacy and numeracy decrease with age in adults aged 60 and older in Tanzania. Hearing and visual complaints are common among seniors; they are more prevalent in rural areas and increase the risk of memory complaints. Our findings may inform the development and suitability of neuropsychological tests for seniors and highlight the need for policymakers to develop dementia prevention interventions and improve access to vision and hearing services for this group.

**Clinical trial number:**

Not applicable.

## Background

Dementia is a major public health problem [1], and the third largest contributor to neurological disability-adjusted life years (DALYs) [2]. It currently affects over 57 million people across the world [3]. Of the 57 million people living with dementia globally, over two-thirds live in low- and middle-income countries (LMICs) where over 75% (28.5 million) remain undiagnosed [1, 2]. Recent dementia prevalence estimates in sub-Saharan Africa (SSA) are comparable to those in high-income countries (HIC), and may be rising due to population aging and the high frequency of risk factors for dementia [4]. This change is likely to result in increased costs to persons living with dementia, as well as their families, communities, and health systems, leading to a loss of economic productivity in SSA [2]. The Lancet Commission on Dementia suggests that approximately 50% of dementias can be prevented or delayed in LMICs through dementia prevention interventions, a rate higher than expected in higher-resourced settings [3].

The identification of dementia using valid and reliable measures of cognitive impairment is crucial for the development of effective preventive interventions, treatment, and care plans. Cognitive performance is markedly affected by demographic, educational, and cultural factors. Currently, available validated SSA neuropsychological testing measures, including the Identification for Dementia in Elderly Africans (IDEA) cognitive screen, may be optimal for advanced dementia but are unproven for mild cognitive impairment where more sensitive tools are needed [5]. While a recent review showed that prevalence estimates of dementia in sub-Saharan Africa (SSA) vary widely [6], our comprehensive literature review found no study that utilized census data to estimate standardized rates of subjective memory, hearing, and visual complaints; reviewed literacy and numeracy rates and highlighted their potential clinical and research implications in SSA settings.

We analysed the *2022 Population and Housing Census* data in Tanzania to determine the age-adjusted prevalence of subjective memory, hearing, and visual complaints and the factors that may influence the development and suitability of neuropsychological assessment batteries among older adults in Tanzania. Additionally, we elucidated the pathways by which age, education, and gender interact to influence subjective memory complaints among seniors.

## Methods

### Study design

This is a secondary analysis of data collected in the *2022 Population and Housing Census* in Tanzania conducted by the National Bureau of Statistics (NBS) in collaboration with other stakeholders [7]. Detailed description of the Country profile (geography, area and population, climate, culture, administrative areas, economy, and currency) has been reported elsewhere [8]. Before data collection, cartography (using Arc-GIS and Global Positioning System technologies) was performed to delineate the entire country into enumeration areas (EAs) [7]. The data used in the present study covered the entire country and were collected from 23rd August 2022 to 29th August 2022 [7].

### Study Population

In the present study, we abstracted data for adults aged 60 years and older.

### Development of the data collection instruments

The Census instruments in digital format, were developed following the United Nations Principles and Recommendations for the 2020 Round of Population Census in collaboration with various stakeholders [7]. The draft questionnaires were reviewed by various stakeholders and the Census Committees; and were pre-tested in 18 regions one year before the actual census and final questionnaires were approved by Central Census Committee [7]. We extracted data for the adults aged 60 years and older including sex, marital status, education level, literacy and numeracy, self-reported or proxy-reported cognitive and sensory symptoms, and employment status (Table 1). While objective assessments of sensory and cognitive functioning are the gold standard for identifying pathological sensory and cognitive changes, self-reported and proxy-reported cognitive complaints in some older adults may serve as preclinical markers of neurodegenerative disease and may reliably measure persistent trends in subjective cognitive complaints (SCCs) over time [9, 10]. They can provide a cost-effective approach to assessing sensory and cognitive functioning and are useful for screening individuals who need further evaluation [10].

**Table 1:** Survey items used to assess subjective sensory and cognitive complaints and other variables analysed in the present study

Variables	Question
Literacy	Can (Name) read and write a short sentence in Swahili, English or Swahili and English or any other language?
Numeracy	Does (Name) have counting skills in Swahili, English or English and Swahili or any other language?
Hearing	Does (Name) have difficulty hearing even if using hearing aid?
Vision	Does (Name) have difficulty seeing even if wearing glasses?
Memory	Does (Name) have difficulty remembering?
Education	What level of education has (NAME) completed or currently attending?
Insurance	Does (Name) have National Health Insurance card, Community Health Insurance or elderly treatment card?
Economic activity	Last week before the Census night which of the following work/activity did [NAME] do for many hours?
Employment	Even though [NAME] did not work last week, did [NAME] have a paid job, or any kind of business, or farming or other activity to generate income that you were absent from and definitely you will return to?

### Recruitment and training of enumerators and supervisors

A total of 205,000 enumerators and supervisors were recruited and trained according to the approved procedures (training of Trainers at national and regional levels and training of supervisors and enumerators at district level). The training covered theoretical and practical aspects of the contents of the questionnaires, interview techniques, and the use of tablets for data collection [7].

### Data collection

Data were collected by enumerators and supervisors, some of whom were positioned at mass transit points and other areas to collect data on travellers and the homeless [7]. Data were submitted to supervisors daily, and submission to the central server at NBS was made by supervisors after performing quality checks following standards and procedures [7]. In the present study, the data file obtained was inspected for completeness and cleaned before analysis. Most of the variables included in this study had < 1% of the data missing, and the missing data was excluded during analysis.

### Statistical analyses

To estimate the age-standardized prevalences of subjective hearing, visual, and memory complaints, the total number of cases within each age stratum was summed to obtain the total number of cases. The total number of cases was divided by the corresponding total population of adults aged 60 and over to ascertain the crude prevalence of these complaints. The age-adjusted prevalences for subjective hearing, visual, and memory complaints were determined based on the World Health Organization (WHO) standard population using the Direct Method. The expected number of cases for each stratum was computed by multiplying stratum-specific prevalence by the size of the stratum in the WHO standard population. The figures were then summed to obtain the total expected cases, which were then divided by the total WHO standard population of adults aged 60 and older to get the directly age-standardized prevalence estimates.

All analyses were performed using SPSS version 29 (IBM, New York, USA) and Jamovi (The jamovi project, 2024) [11]. All continuous data were non-normally distributed and therefore were analysed using non-parametric statistical tests. Pearson’s Chi-square test was used to examine the association between categorical variables and subjective memory complaints. The 95% confidence intervals for prevalence estimates were computed assuming a binary distribution and using the corresponding total population of adults aged 60 and older as a denominator.

Variables with *p* < 0.05 were entered into bivariate logistic regression models to determine their association with subjective memory complaints. Multivariable logistic regression models were constructed to determine the association between the independent factors and subjective memory complaints. To ascertain the accuracy of the models, we examined the goodness of fit, the coefficients of determination, and the studentized residual values. Mediation analysis was conducted using path analysis to estimate the effects of age, gender, and education on memory complaints and the significance of the indirect effects was tested using bootstrapping procedures. A prior significance was set at *p* < 0.05 for all statistical tests.

## Results

### Comparison of sociodemographic characteristics of older adults between rural and urban areas

A total of 349,674 older adults were included in the analysis with a median (IQR) age of 68 (60– 97, Table 2). Most adults were female (*n* = 190,691, 54.5%); married or living together (198,785, 56.8%), and resided in rural areas (*n* = 250,760, 71.7%). While significant differences in distribution by age were noted between rural and urban areas (*p* ≤ 0.001), sex distribution by region did not differ (*p* = 0.127). Conversely, the distribution of older adults by marital status and health insurance was significant, with most married adults and those without health insurance living in rural areas (*p* ≤ 0.001). The rural areas had more seniors engaged in economic activities and those self-employed without workers (*p* ≤ 0.001).

**Table 2:** Comparison of sociodemographic characteristics of older adults based on place of residence (*n* = 349,674)

Variables	Rural *N* (%)	Urban *N* (%)	*P* value
**Age**: Md = 68 (IQR:60,97)			
60– 64	80,058 (32.0)	35,301 (35.7)	≤ 0.001
65–69	49,268 (19.6)	21,341 (21.6)	
70– 74	45,642 (18.2)	17,404 (17.6)	
75–79	27,572 (11.0)	9,794 (9.9)	
80–84	21,872 (8.7)	69,43 (7.0)	
≥ 85	26,348 (10.5)	8,131 (8.2)	
**Sex**			
Female	136,952 (54.6)	53,739 (54.3)	0.127
**Marital Status**			
Never Married	9376 (3.7)	4,421 (4.5)	≤ 0.001
Married/Living together	145,346 (58.0)	53,439 (54.0)	
Widowed	22,938 (9.1)	9,666 (9.8)	
Divorced/Separated	73,100 (29.2)	33,388 (31.7)	
Health Insurance*			
Yes	4,145 (1.7)	2,300 (2.3)	≤ 0.001
Economic activity*			
Yes	141,801 (56.5)	51,038 (51.6)	≤ 0.001
Employment Status*			
Paid Employee	394 (8.4)	155 (11.0)	≤ 0.001
Self-employment without workers	2942 (63.0)	750 (53.3)	
Self-employment with workers	491 (10.5)	242 (17.2)	
Unskilled labour	600 (12.8)	177 (12.6)	
Unrecognized employee	243 (5.2)	83 (5.9)	

* There was missing data on health insurance, economic activity and employment status

### Educational level, literacy and numeracy rates among older adults in Tanzania

An estimated 59.7% (*n* = 208,815) of older adults were literate, of whom 57.5% (*n* = 120,123) were male (Table 3**).** Of the 67.7% (*n* = 234,416) older adults who were numerate, 53.3% (*n* = 124,916) were male. Literacy and numeracy rates varied significantly across genders (*p* ≤ 0.001). The Gender Parity Index (GPI) was 0.89 indicating that female older adults were 11% less likely to be literate compared to their male counterparts.

Literacy and numeracy rates decreased with age, with most literate and numerate older adults residing in urban areas (*p* ≤ 0.001). While female older adults comprised the majority of older adults with an education level of less than 7 years (52.4%), male older adults comprised the greater proportion of older adults with education levels between 7 and 11 years (62.6%); 12–16 years (69.4%); and 17 years and over (69.2%). The urban population had a smaller proportion of adults with an education level of less than 7 years and a significantly larger proportion of adults with other categories of educational attainment compared to the rural population (*p* ≤ 0.001).

**Table 3:** Comparison of educational level, literacy, and numeracy rates among older adults based on place of residence (*N* = 349,674)

Variables	Rural *N* (%)	Urban *N* (%)	Significance (*p* value)
Literacy rates			
**Age band**			
60– 64	51,363 (64.2)	29,687 (84.1)	≤ 0.001
65–69	28,857 (58.6)	17,196 (80.6)	
70– 74	23,567 (51.6)	12,857 (73.9)	
75–79	12,932 (46.9)	6673 (68.1)	
80–84	8,934 (40.8)	4,098 (59.0)	
≥ 85	8,591 (32.6)	4060 (49.9)	
**Sex**			
Female	53,931(40.2)	34,761 (46.6)	≤ 0.001
Male	80,313 (59.8)	39,810 (53.4)	
**Numeracy rates**			
** Age band**			
60– 64	58,512 (73.1)	29,645 (84.0)	≤ 0.001
65–69	34,056 (69.1)	17,411 (81.6)	
70– 74	28,677 (62.8)	13,146 (75.5)	
75–79	15,966 (57.9)	6,874 (70.2)	
80–84	11,166 (51.1)	4,291 (61.8)	
≥ 85	10,645 (40.4)	4.027 (49.5)	
**Sex**			
Male	72,928 (45.9)	36,572 (48.5)	≤ 0.001
Female	86,094 (54.1)	38,822 (51.5)	
**Education level**			
Less than 7 years	51,086 (41.7)	16,726 (25.1)	≤ 0.001
7 to 11 years	63,498 (51.8)	35,307 (53.1)	
12 to 16 years	5,105 (4.2)	8,422 (12.6)	
17 years and above	2,850 (2.3)	6,158 (9.2)	

### Frequent cognitive and sensory complaints among older adults in Tanzania

The subjective memory complaints increased with age (Table 4). The crude prevalence of subject memory complaints was 7.9% (95%CI: 7.9–8.1) while the age-adjusted prevalence was 7.3 (95%CI: 7.2–7.4). Age-adjusted prevalence of subjective memory complaints was higher in female older adults (8.1%; 95%CI: 8.0–8.2) than in male older adults (5.7%; 95%CI: 5.6–5.8) and in rural (7.1%; 95CI:7.0– 7.2) than in urban settings (6.8%; 95%CI: 6.6–6.9).

**Table 4:** Prevalence of subjective memory complaints among older adults

Age group	Stratum population	Cases	Stratum specific Proportion	Size of stratum in WHO population	Estimated cases
60–64	115,359	3,819	0.03	372,000,000	12,315,190
65–69	70,609	3,344	0.05	292, 000,000	13,828,945
70–74	63,046	4,325	0.07	221,000,000	15,160,756
75–79	37,366	4,278	0.11	152,000,000	17,402,344
80–84	28,815	4,127	0.14	91,000,000	13,033,385
85+	34,479	7,702	0.22	63,000,000	14,073,088
**TOTAL**				1,191, 000,000	85,813,709
Crude prevalence of subjective memory complaints (95%CI)					7.9 (7.8– 8.0)
Age-adjusted prevalence of subjective memory complaints (95%CI)					7.2 (7.1–7. 3)

**Fig. 1:**
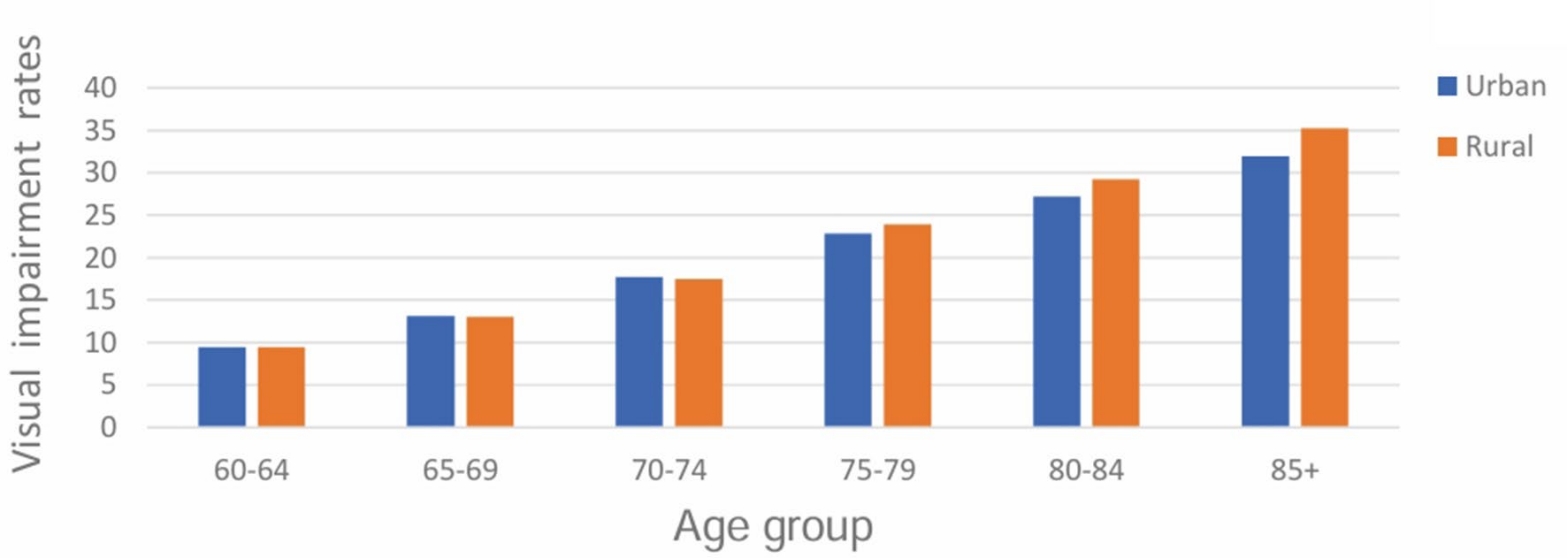
Visual complaints significantly increased with age (χ² [5, *N* = 346,542] = 16,655.67; *p* ≤ 0.001) and were higher in rural compared to urban settings (χ² [1, *N* = 346,542] = 109.97; *p* ≤ 0.001)

**Fig. 2:**
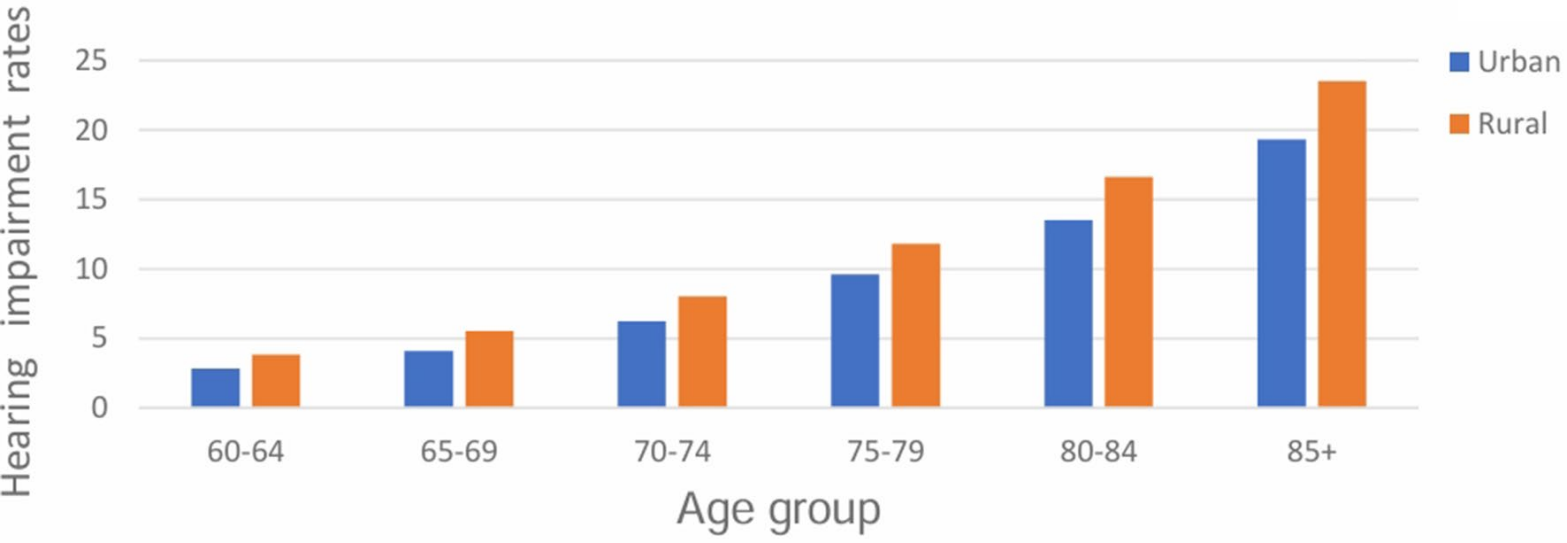
Hearing complaints were higher among older adults in rural areas compared to urban areas (χ² [1, *N* = 346,542] = 579.21; *p* ≤ 0.001)

Both visual and hearing complaints significantly increased with age and were higher in rural compared to urban settings (Fig. 1 and Fig. 2). The crude and age-adjusted prevalence estimates of hearing complaints in older adults aged 60 + were 8.2% (95%CI: 8.1–8.3) and 7.5% (95%CI: 7.4–7.6) respectively. Age-adjusted prevalence of hearing complaints was higher in female older adults (8.4%; 95%CI: 8.3– 8.5) compared to male older adults (6.4%; 95%CI: 6.3–6.5). Crude and age-adjusted visual complaints were 17.1% (95%CI: 16.9–17.2) and 16.3% (95%CI: 16.2– 16.4) respectively. Age-adjusted prevalence of visual complaints was higher in female older adults (17.1%; 95%CI: 16.9 − 17.2) compared to male older adults (15.4%; 95%CI:15.3–15.6).

### Factors associated with subjective memory complaints among older adults: bivariate logistic regression

The bivariate logistic regression analysis revealed significant associations between age, sex, marital status, literacy, hearing complaints, visual complaints, and education level (Table 5). Compared with adults aged 60–64, those aged 65 and over had a significantly increased likelihood of subjective memory complaints, with the highest increase occurring in the 85 + age group (*p* ≤ 0.001). Likewise, female older adults (OR = 1.37; 95%CI: 1.30–1.44; *p* ≤ 0.001), older adults with hearing complaints (OR = 7.12;95%CI: 6.78–7.47; *p* ≤ 0.001), and those with visual complaints (OR = 7.12; 95%CI: 6.78–7.47; *p* ≤ 0.001) had significantly increased odds of subjective memory complaints. Conversely, older adults who were married or living together (OR = 0.83; 95%CI: 0.74–0.93; *p* = 0.002), older adults who were literate (OR = 0.59; 95%CI: 0.57–0.60; *p* ≤ 0.001) and those with a 7-year education level and higher(*p* ≤ 0.001), had significantly reduced odds of subjective memory complaints.

**Table 5:** Factors associated with subjective memory complaints among older adults: bivariate logistic regression

Variable*	Proportion, *N* (%)	B^†^	OR (95%CI)	Significance(*p*-value)
**Age**				
60–64	3,819 (14.1)	1.00	1.00	----
65–69	3,344 (12.3)	0.22	1.24 (1.16–1.33)	≤ 0.001
70–74	4,325 (16.0)	0.43	1.54 (1.43–1.65)	≤ 0.001
75–79	3,778 (13.9)	0.64	1.90 (1.75–2.06)	≤ 0.001
80–84	4,117(15.2)	0.85	2.35 (2.15–2.56)	≤ 0.001
85+	7,702 (28.4)	1.20	3.34 (3.06–3.64)	≤ 0.001
**Sex**				
Male	9,460(34.9)	1.00	1.00	---
Female	17,625 (65.1)	0.31	1.37 (1.30–1.44)	≤ 0.001
**Marital status**				
Never married	1,175 (4.3)	1.00	1.00	---
Married/Living together	10,661(39.4)	-0.19	0.83 (0.74–0.93)	0.002
Divorced/Separated	2,399 (8.9)	0.10	1.01 (0.88– 1.16)	0.882
Widowed	12,850 (47.4)	0.40	1.04 (0.92– 1.17)	0.540
**Literate**				
No	14,232 (52.5)	1.00	1.00	---
Yes	12,853 (47.5)	-0.53	0.59 (0.57–0.60)	≤ 0.001
**Visual complaints**				
Yes	17,361(64.1)	1.96	7.12 (6.78–7.47)	≤ 0.001
No	9,724 (35.9)	1.00	1.00	-----
**Hearing complaints**				
Yes	12,264 (45.3)	1.53	4.62 (4.37–4.88)	≤ 0.001
No	14,821 (54.7)	1.00	1.00	----
**Bilingual**				
Yes	1,309 (10.2)	0.04	1.04 (0.96–1.13)	0.317
No	11,544 (89.8)	1.00	1.00	----
**Education level**				
Less than 7 years	6,063 (54.0)	1.00	1.00	----
7 to 11 years	4,348 (38.7)	-0.76	0.47 (0. 45 − 0.49)	≤ 0.001
12 to 16 years	463 (4.1)	-1.01	0.36 (0.33–0.40)	≤ 0.001
17 years and above	351 (3.1)	-0.86	0.42 (0.38– 0.47)	≤ 0.001

^†^B: coefficient of regression. *There was missing data on all variables

### Factors associated with subjective memory complaints among older adults: multivariable logistic regression

Multivariable logistic regression models containing six variables (age, sex, marital status, hearing and visual complaints, and education level) were constructed (Table 6). All variables contributed significantly to the model. The odds of memory complaints increased significantly with older age, with notable effects commencing in the 65–69 age band (AOR = 1.24; 95%CI: 1.16–1.33; *p* ≤ 0.001) and becoming substantially pronounced in the 85 + age band (AOR = 3.34; 95%CI:3.06–3.64; *p* ≤ 0.001). While the odds of subjective memory complaints were higher in female older adults (AOR = 1.37; 95%CI:1.30–1.44; *p* ≤ 0.001) compared with male older adults, older adults who were married or living together (AOR = 0.83; 95%CI: 0.74–0.93; *p* = 002) and those who attained a 7-year education level and higher (p = ≤ 0.001), had significantly reduced odds of subjective memory complaints. The odds of subjective memory complaints were more than 4 and 7 times higher for older adults with hearing (AOR = 4.61; 95%CI: 4.36–4.88; *p* ≤ 0.001) and visual (AOR = 7.12; 95%CI: 6.78–7.47: *p* ≤ 0.001) complaints respectively compared with their counterparts.

**Table 6:** Factors associated with subjective memory complaints: multivariable logistic regression

Variable*	Proportion, *N* (%)	B^†^	AOR^‡^ (95%CI)	Significance(*p*-value)
**Age**				
60–64	3,819 (14.1)	1.00	1.00	----
65–69	3,344 (12.3)	0.22	1.24 (1.16–1.33)	≤ 0.001
70–74	4,325 (16.0)	0.43	1.54 (1.43–1.65)	≤ 0.001
75–79	3,778 (13.9)	0.64	1.90 (1.75–2.06)	≤ 0.001
80–84	4,117 (15.2)	0.86	2.35 (2.15–2.56)	≤ 0.001
85+	7,702 (28.4)	1.21	3.34 (3.06–3.64)	≤ 0.001
**Sex**				
Male	9,460 (34.9)	1.00	1.00	---
Female	17,625 (65.1)	0.31	1.37 (1.30–1.44)	≤ 0.001
**Marital status**				
Never married	1,175 (4.3)	1.00	1.00	---
Married/Living together	10,661 (39.4)	-0.19	0.83 (0.74–0.93)	0.002
Divorced/Separated	2,399 (8.9)	0.01	1.01 (0.88– 1.16)	0.884
Widowed	12,850 (47.4)	0.04	1.04 (0.92– 1.17)	0.542
**Visual Complaints**				
Yes	17,361(64.1)	1.96	7.12 (6.78–7.47)	≤ 0.001
No	9,724 (35.9)	1.00	1.00	-----
**Hearing complaints**				
Yes	12,264 (45.3)	1.53	4.61 (4.36–4.88)	≤ 0.001
No	14,821 (54.7)	1.00	1.00	----
**Education level**				
Less than 7 years	6,063 (54.0)	1.00	1.00	----
7 to 11 years	4,348 (38.7)	-0.09	0.91 (0. 87 − 0.96)	≤ 0.001
12 to 16 years	463 (4.1)	-0.29	0.75 (0.66– 0.84)	≤ 0.001
17 years and above	351 (3.1)	-0.16	0.85 (0.74–0.97)	0.019

^†^B: coefficient of regression; ^‡^AOR: adjusted odds ratio

### Mediators of subjective memory complaints among older adults: mediation analysis

**Fig. 3:**
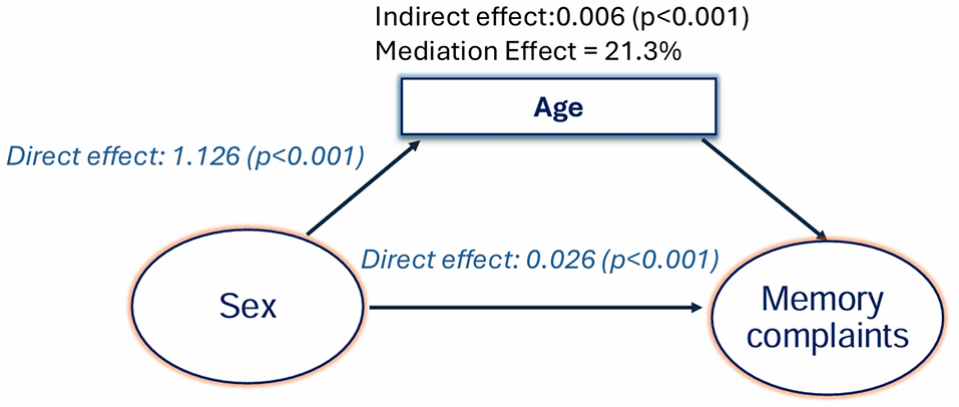
Mediation effects with age as a mediator for sex: age accounted for 21% of the effect of sex on subjective memory complaints

**Fig. 4:**
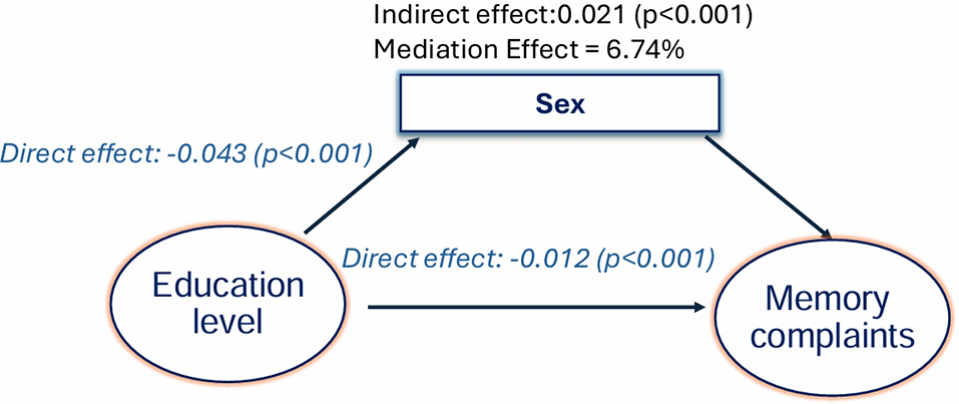
Mediation effects with sex as a mediator for education: Female sex accounted for about 7% of the effect of education on subjective memory complaints

Mediation analysis indicates significant interaction effects between age, sex, and education on subjective memory complaints (Figs. 3 and 4). As Fig. 3 illustrates, the effect of sex on subjective memory complaints was mediated by age; age accounted for 21% of the effect of sex on subjective memory complaints (*p* ≤ 0.001). With sex as a mediator, the effect of education on memory complaints was lower for women (Fig. 4). While education accounted for 93% of the effect of education on subjective memory complaints directly, being female reduced the effect of education on subjective memory complaints by 7% (*p* ≤ 0.001).

## Discussion

The strength of our study in SSA involves the use of census data to estimate standardized rates of subjective hearing, visual, and memory complaints, review literacy, and numeracy rates, and highlight their potential clinical and research implications in SSA settings. We found that hearing and visual complaints are highly prevalent in this age group; increase with age, and increase the likelihood of subjective memory complaints. Additionally, our study showed that literacy and numeracy rates are lower in this group, higher in urban compared to rural areas, and decrease with older age. We further demonstrated that age lowers the effect of gender on subjective memory complaints while female sex lowers the effect of education on subjective memory complaints.

In the current study, we found that the age-adjusted prevalence of hearing complaints was higher in female older adults (8.4%; 95%CI: 8.3– 8.5) compared to male older adults (6.4%; 95%CI: 6.3–6.5). Comparing our findings on the prevalence of hearing complaints with those of other studies may be problematic due to the lack of conformity in assessment methods used [12–14] (self-reported vs. audiometric assessment), selection of the study population [14–16] (older adults aged 60 + vs. older adults aged 70+), study designs [12, 13, 17] (cross-sectional vs. longitudinal), the severity of hearing problems [12, 13, 17] (self-rated vs. objective), study setting [18–21] (hospital vs. community), case definition [12, 16] (tonal frequencies and audiometric threshold used) and type of hearing loss [17, 21] (conductive vs. sensorineural). Notwithstanding the limitations, our prevalence estimates of hearing complaints are consistent with WHO prevalence estimates of hearing loss of moderate or higher grade among adults aged 60–69 years (10.9–17.6%) across the WHO regions and the global prevalence rate of hearing loss of 7.5% in HICs [17]. A recent systematic review on sex differences in auditory function involving eleven studies concluded that pre-menopausal women have better hearing compared to men, while female older adults have a reduction in hearing sensitivity compared to age-matched men [22], which may explain the higher prevalence of hearing complaints observed in female older adults in this study. Although sex-related differences in auditory function may be due to increased longevity in women, noise exposure, ototoxicity, and changes in female sex hormones [22], the paucity of high-quality research in this area underscores the need for further research to unravel inherent sex-related mechanisms underlying auditory perception that could explain the observed sex-based differences in hearing loss in older adults [22].

As with studies on hearing loss, variability resulting from non-homogeneous study designs [23], selection of study population [23] evaluation methods, study settings, and severity [23] make comparison of our findings on visual complaints challenging. Nevertheless, our age-adjusted prevalence of visual complaints largely aligns with the prevalence of moderate and severe visual impairment reported by the 2017 Systematic review and meta-analysis in South Asia (17·5%, 80% UI 9·1–27·2), North America (17·5%, 80% UI 9·1–27·2), the Middle East (17·2%, 8·6–26·8), western sub-Saharan Africa (16·0%, 8·0–25·3), central sub-Saharan Africa (14·4%, 6·3–24·5), and Southeast Asia (14·1%, 6·9–22·3) in older adults aged 50 years and older [24].

We found a higher prevalence of visual complaints among female older adults and a greater proportion of seniors with visual complaints in rural areas, in line with the overall trend observed in previous research [25]. While this difference in vision impairment has been attributed to barriers related to the availability, accessibility, acceptability, and affordability of eye care services [25], the recent finding that males significantly outperformed females in visual tasks [26], highlights the need for further research to understand the sex-related neurodevelopmental, genetic, epigenetic, hormonal and neurophysiological mechanisms that may account for differential susceptibility to vision impairment between males and females.

Inequalities in literacy and education attainment between male and female older adults; and between rural and urban settings noted in the present study are widely documented [27] and can be explained by social determinants of education that impact the availability, accessibility, and affordability of quality education [28]. Our findings support the existence of cohort differences in education which may suggest differential learning experiences and dramatic improvements in education occurring across successive cohorts [27]. Intuitively, it is plausible that cohort differences may indicate inherent learning experiences and cognitive abilities should be appraised when developing and selecting neuropsychological tests for this cohort. Consequently, a one-size-fits-all neuropsychological assessment battery may be problematic in this age group as they may need a cohort-specific, culturally and contextually relevant neuropsychological assessment battery.

Comparing our results on subjective memory complaints with previous studies is fraught with methodological difficulties. The same is true for the findings on hearing and vision impairments above. However, our findings are consistent with prevalence estimates of subjective cognitive complaints (SCD) reported in other studies [29]. A recent study on subjective cognitive complaints across 16 population cohorts (*n* = 39,387) reported age- and gender-standardized prevalence estimates for subjective cognitive decline (SCD) ranging from 6.1% (95% CI = 5.1–7.0%) to 52.7% (95% CI = 47.4–58.0%), utilizing a qualitative harmonization (QH) strategy [29]. In contrast to our findings, this same study reported a pooled prevalence estimate of SCD at 24% among adults aged 60 and older. This discrepancy may stem from the fact that most cohorts included in the study were not based in African nations and comprised older groups, with a mean age of 73.1 years (SD = 7.1; range = 60–105 years [29]. Additionally, a meta-analysis of 31 studies from 15 countries in Latin America reported a higher prevalence of dementia in females than in males and in rural than in urban settings [20]. Our results on marital status and subjective memory complaints mirror those of recent research which reported a reduced risk of cognitive impairment in married seniors compared to their unmarried counterparts [30]. It is hypothesized that married persons may have greater access to economic resources (access to health services, nutrition, and other amenities), institutional memory (when spouses remind them of important events and reflect on past experiences), and social resources (social support, and social integration) which enhance cognitive reserve and protects against cognitive impairment [30].

Our findings on advanced age, female sex, and low education conform to previous research, which demonstrated that advanced age [31], female sex [3], and low education [3] are associated with cognitive impairment. The effect of age on cognitive impairment can be explained by age-related biological [32] and social factors [33] that contribute to progressive neuronal loss and disruption of brain networks critical for cognitive function. While sex differences in cognitive impairment in older adults are attributed to longer life spans and lower educational attainment in women [3], other inherent sex-based mechanisms [32] (changes in sex hormone signalling, risk genes, immune responses) and variations in dementia risk factors [34] (including cardiovascular risk factors, depression, and physical activity) may be significant risk determinants for older women [3].

Our findings on hearing complaints are largely consistent with previous studies which found that hearing loss increases the likelihood of cognitive impairment [3, 16]. Researchers postulate that psychosocial factors [35], reduced cognitive reserve stemming from reduced environmental stimulation [3], and common cardiovascular pathology [3, 36] may explain the increased risk of dementia in older adults with hearing loss. Consistent with other studies [3, 23], we found an increased likelihood of subjective memory complaints in seniors with vision impairment. Proposed mechanisms to explain how visual impairment increases the risk of dementia include shared neuropathology [23], common pathophysiology [37], and the impact of visual loss on visual cognition and perception [38, 39].

Our study indicates significant potential clinical and research implications with regard to the development and selection of neuropsychological tests for seniors. The effects of education are consistently demonstrated in tests of cognitive abilities, including those deemed relatively unaffected by years of formal education, such as simple drawing tasks, leading to a misinterpretation of the effects of low education as cognitive impairment [40]. Educational attainment, career achievements, sociodemographic factors, and cultural influences contribute to cognitive reserve, and individuals with higher cognitive reserves may perform in the normal range despite being impaired [40]. Limited literacy can profoundly influence the development of neurocognitive abilities, processing pathways, strategies, and the functional organization of the brain [41]. Non-literate persons and those with low education typically perform poorly on tasks involving reading, writing, arithmetic, drawing, praxis, visuospatial, and visuo-constructive skills [40, 41]. The higher literacy and numeracy rates at the lower age band in the present study suggest that the proportion of individuals needing non-literate-specific assessments will diminish over time, notably in urban areas. Additionally, most of the existing tests require intact hearing and vision and may present significant administration and validity challenges since few if any have been validated in adults with hearing and vision impairment in Tanzania.

Overall, clinicians and researchers should carefully consider these subgroups of adults: the non-literate, the monolingual individuals, adults with different educational attainment, and those with hearing or visual impairments, along with their potential ramifications when developing and selecting neuropsychological tests for this population. The development and selection of neuropsychological tests should be tailored to accommodate adults with low literacy levels and sensory impairments. Validating test batteries with minimal educational and linguistic demands and those validated for persons with visual impairment in other settings may be a viable approach [42, 43]. The high prevalence of modifiable risk factors for dementia in this population highlights the need for policymakers and other stakeholders to create dementia prevention interventions and enhance access to vision and hearing services.

While the present study provides valuable insights into risk factors for dementia and their impact on the selection and development of neuropsychological batteries among seniors, it is crucial to consider several limitations when interpreting our findings.

Although the present data provides strong statistical power and broad applicability, the large census dataset raises the possibility that small, insignificant effects could appear statistically significant. To mitigate the risk of obscuring subtle subgroup variations inherent in census data, we utilized a representative 10% of the dataset and performed separate analyses for adults aged 60 and older. Despite this, we cannot exclude masking such variations in this study due to the large sample size.

Although enumerators were trained to probe literacy, hearing, and visual impairments, no objective measures were utilized. As seniors may be unaware of their cognitive and sensory problems, the census likely captured those with striking impairments. This could lead to an underestimation of our prevalence estimates.

The cross-sectional nature of our study limits our ability to establish causal relationships between the factors and subjective memory complaints. Additionally, cognitive and sensory symptoms were based on self-reports, which could be influenced by sex (women may report cognitive symptoms more frequently than men), culture (some seniors may deny or underreport cognitive impairment due to stigma), and educational biases (less educated individuals often misreport their memory) [44]. Longitudinal research incorporating objective measures of hearing and vision along with neuropsychological tests or biomarkers may complement self-reported cognitive impairment, mitigate the impact of subjective biases, and provide richer insights into potential aetiologies and causal relationships between these factors and memory impairment in this population.

In conclusion, literacy and numeracy decrease with increasing age in those over 60. Hearing and visual complaints are predominant among seniors; they are more prevalent in rural areas and increase the risk of memory complaints. Our findings may inform the development and selection of neuropsychological batteries for seniors and highlight the need for policymakers to develop dementia prevention interventions and improve access to vision and hearing services for this population.

## Data Availability

The data related to our manuscript is publicly available on the website of the National Bureau of Statistics at the following URLhttps://www.nbs.go.tz.
